# Optimizing calibration designs with uncertainty in abilities

**DOI:** 10.1111/bmsp.12387

**Published:** 2025-03-10

**Authors:** Jonas Bjermo, Ellinor Fackle‐Fornius, Frank Miller

**Affiliations:** ^1^ Department of Computer and Information Science Linköping University Linköping Sweden; ^2^ Department of Statistics Stockholm University Stockholm Sweden

**Keywords:** ability, computerized adaptive tests, item calibration, optimal experimental design

## Abstract

Before items can be implemented in a test, the item characteristics need to be calibrated through pretesting. To achieve high‐quality tests, it's crucial to maximize the precision of estimates obtained during item calibration. Higher precision can be attained if calibration items are allocated to examinees based on their individual abilities. Methods from optimal experimental design can be used to derive an optimal ability‐matched calibration design. However, such an optimal design assumes known abilities of the examinees. In practice, the abilities are unknown and estimated based on a limited number of operational items. We develop the theory for handling the uncertainty in abilities in a proper way and show how the optimal calibration design can be derived when taking account of this uncertainty. We demonstrate that the derived designs are more robust when the uncertainty in abilities is acknowledged. Additionally, the method has been implemented in the R‐package optical.

## 1 INTRODUCTION

Test items are typically pretested and stored in an item bank before being used in an operational test. The operational test may be either a fixed‐form test, where all examinees get the same set of items, or a computerized adaptive test (CAT), where items are selected adaptively based on the current ability estimate of each examinee. Continuously replenishing the items in the bank is essential as the items get overexposed or obsolete over time, especially for CAT. The procedure of estimating the item parameters such as difficulty and discrimination is referred to as item calibration. Ensuring the precise estimation of the items' parameters is crucial, as the accuracy of these parameters impacts the precision of the latent ability estimates (Cheng & Yuan, 2010; Tsutakawa & Johnson, 1990; Zhang et al., 2011) of future test takers. This is of particular importance for CAT (Ali & Chang, 2014), because examinee ability estimates are continuously updated during the test and items are chosen to match current estimates.

Different calibration strategies exist: the calibration items can be integrated into an operational test or given as a separate test. If the test is computerized, the assignment of items to examinees can be done adaptively. Item calibration can be performed either ‘online’ or ‘offline’. With ‘online’ calibration, the item parameters are continuously estimated in real time during a CAT (Stocking, 1988), whereas with ‘offline’ calibration parameter estimation is performed after the test is completed (He et al., 2020).

The item parameter estimates depend on the unknown latent abilities of the examinees participating in the calibration. However, we only have access to abilities being estimated with some uncertainty. The calibration methods differ in whether they explicitly account for this uncertainty in ability estimates during item parameter estimation. For example, Stocking's Method A treats estimated abilities as true abilities (Stocking, 1988), whereas the marginal maximum likelihood estimate (MLE) with one expectation‐maximization (OEM) cycle and the marginal MLE with multiple expectation‐maximization (MEM) cycles model this uncertainty (Ban et al., 2001; Chen et al., 2012, 2017; Chen & Wang, 2016). Ban et al. (2001) performed a comparative study of five methods and concluded that the MEM method is the best choice for item parameter recovery when compared to OEM, Methods A and B (Stocking, 1988) and the BILOG/Prior method (Mislevy & Bock, 1990).

Methods of optimal design can be applied in an item calibration context to determine which examinees to select from a population such that the item parameters are estimated with as high precision (low variance) as possible. The precision of the item parameter estimates is dependent on the ability of the respondent. How to select a sample of examinees with the most appropriate abilities can be treated as an optimal design problem (Berger, 1991; Buyske, 2005).

The approach employed for determining an optimal design depends on the test setting in which the calibration is performed. In an adaptive test, examinees arrive sequentially to be assigned the next item based on their most recently estimated ability, as in for example the Programme for International Student Assessment (OECD, 2024), whereas in a parallel test, a large number of examinees take the test simultaneously, as in for example the Swedish Scholastic Aptitude Test (SweSAT; Umeå University, 2022).

Ul Hassan and Miller (2019) developed a method to derive optimal designs to allocate items to examinees in a non‐adaptive testing setting. The method derives ability intervals that dictate the assignment of items to examinees based on their ability level. Each examinee is assigned items in accordance with the interval in which their ability falls. When deriving the ability intervals, the abilities θ are assumed to be known. Therefore, before the optimal design method is employed, the examinees' abilities need to be estimated from operational items. One option is the integrated set‐up, with calibration items being incorporated into an operational test, typically positioned towards the end of the test but in a way that the examinees cannot distinguish calibration from operational items (Ren et al., 2017; Stocking, 1988). Such a set‐up is, e.g.  used in the Swedish driving license test, where 5 of 70 items serve as calibration items (Korkortonline.se, 2024). Another option is the separated set‐up, where a test consists exclusively of calibration items. In this set‐up, examinees may have previously taken a distinct operational test. This is the set‐up for the Swedish national tests in mathematics (Skolverket, 2023), where the calibration test occurs weeks or months after the operational test for a subset of examinees who participated in the operational test. Irrespective of whether an integrated or separated set‐up is chosen for calibration, it is assumed that estimated abilities $\hat{\theta }$ are available for each examinee participating in the calibration part of the test. Bjermo et al. (2024) applied this method using data from the SweSAT.

The method of Ul Hassan and Miller (2019) assumes known abilities when deriving the optimal designs, but the fact that we only have access to estimated abilities may affect the optimal assignment of calibration items. The main objective of this paper is, therefore, to account for the uncertainty of the estimated abilities $\hat{\theta }$ and to adjust the optimal design method to adequately handle the uncertainty.

The optimal Bayesian adaptive design approach of van der Linden and Ren (2015) uses the posterior distribution of the individual abilities and thereby takes the uncertainty into account. Their optimal design approach is performed sequentially and optimizes the items for the next examinee in a CAT setting. In contrast, our approach determines the optimal calibration item allocation for the entire group of examinees simultaneously. Our approach is particularly suited for testing scenarios involving large groups of examinees being tested in parallel, such as the Swedish national tests or the SweSAT.

To deal with abilities being estimated, we need to quantify the uncertainty of $\hat{\theta }$. We will mainly investigate the case where we can assume an asymptotic distribution of the abilities that is dependent on the item parameters in the operational test and the estimated abilities. Additionally, we explore a method that does not rely on any assumptions about the distribution of θ. Based on the estimated abilities and their quantified uncertainties, we derive optimal uncertainty‐adjusted calibration designs.

The article is organized in the following way. We begin by describing the calibration set‐up considered in this paper. We then outline the optimal calibration design method by Ul Hassan and Miller (2019). Following this, we derive the theory for handling uncertainty in ability estimates and the associated adjustments to the information matrix. We describe how to calculate the relative efficiency and show how the results can be extended to other models. Additionally, we describe how to evaluate the uncertainty‐adjusted design methodology. We calculate the standardized information matrix for three different cases and conduct a comparative analysis. We continue with some examples of calibration blocks with two, three and four items. These examples illustrate how our method of uncertainty‐adjusted optimal design works in different scenarios. Then, we compare the efficiencies to a random design (RD) and describe a small simulation study for examine bias and mean squared error (MSE). We also describe how to get the results in the paper from the R‐package optical. All results are presented and explained in the Results section. Finally, we conclude with a discussion of the proposed method and the results.

## 2 SET‐UP

We assume in this article that we have a total of n items requiring calibration and m operational items that every examinee takes prior to the calibration items. The calibration items can either be integrated into an operational test or two separate tests might be conducted. The aim is to determine how to allocate the examinees to the calibration items based on the estimated abilities from the operational items taking the uncertainty into account. We further assume that anyone in a population of examinees can calibrate exactly one of the calibration items. There is, however, no limitation on the number of examinees that can calibrate one item. If we, e.g., have four calibration items, the method will determine intervals on the ability scale that dictates which one of the four items an examinee will be given. This means that two examinees with the same estimated ability will receive the same item.

Although this assumption may initially appear limiting, once we establish an optimal method for allocating items to examinees, we can readily scale up the approach to real‐world scenarios where each examinee calibrates multiple items, say k. We can do this by grouping items into k blocks such that any examinee is assigned to exactly one item within each block. Subsequently, we will apply the optimal allocation method, which we will discuss in detail in this article, to each individual block; see section 5 of Ul Hassan and Miller (2019). For example, if we have 40 items to calibrate, we can divide them into 10 blocks, each containing 4 items. In this way, each examinee will calibrate 10 items, 1 selected from each block.

The methods developed in Section 3 will differ depending on the number of operational items. With a large enough number of operational items, we can make distributional assumptions that enable the determination of the optimal ability intervals before the operational items are taken. Without that assumption, we need to calculate the optimal ability intervals based on responses from the operational items.

Calculating the optimal design before the results of the operational items are available enables the immediate allocation of the calibration items. On the contrary, when calculating the optimal design after the results of the operational items are available, we cannot assign the calibration items instantly, which might be unsuitable for certain test situations.

## 3 THEORY

### 3.1 Information matrix and design optimization assuming known abilities

We first recapitulate the optimal calibration design method under the assumption of known abilities. Our objective is to calibrate a set of n items, referred to as calibration items. We consider the so‐called sampling design problem, wherein the goal is to identify an optimal allocation rule determining which ability levels should be sampled to calibrate specific items.

The probability for an examinee with ability θ∈Θ=R to correctly respond to item i (i=1,…n) is

$$p_{i}(\theta )=P(Y_{i}=1|\theta ,\zeta_{i}).$$

Here $Y_{i}=1$ indicates a correct response, $Y_{i}=0$ is an incorrect response and $\zeta_{i}$ is the item parameter vector of item i.

For the two‐parameter logistic (2PL) model, the function $p_{i}(\theta )$ has item parameters $\zeta_{i}=(a_{i},b_{i})\in (0,\infty )\times R$ and is defined as

(1) $$p_{i}(\theta )=P(Y_{i}=1|\theta ,a_{i},b_{i})=\frac{1}{1+e^{-a_{i}(\theta -b_{i})}}.$$

The model (1) is a generalized linear model (GLM) with logit link $\eta_{i}(\theta )=\log\left(\frac{p_{i}(\theta )}{1-p_{i}(\theta )}\right)$. Linearization of the non‐linear model means that we consider $\frac{\partial \eta_{i}(\theta )}{\partial \zeta_{i}}$ instead of the design matrix X as for a linear model (Atkinsson et al., 2007).

Following Ul Hassan and Miller (2019), the standardized information matrix of item parameters $\zeta =(\zeta_{1},\text{…},\zeta_{n})$ is a block‐diagonal matrix

$$M(h)=\text{diag}(M_{1}(h_{1}),\text{…},M_{n}(h_{n})),$$

with

$$M_{i}(h_{i})=\int_{\Theta }p_{i}(\theta )(1-p_{i}(\theta ))\left(\frac{\partial \eta (\theta )}{\partial \zeta_{i}}\right){\left(\frac{\partial \eta (\theta )}{\partial \zeta_{i}}\right)}^{T}h_{i}(\theta )d\theta .$$

Here, $h_{i}(\theta )\geq 0$ specifies the design for item i: It is the ability‐subdensity of examinees allocated to item i. The total population of examinees is assumed to have a known ability density $h_{tot}$; therefore, $\sum_{i=1}^{n}h_{i}(\theta )=h_{tot}(\theta )$ for all θ. A reasonable assumption is that $h_{tot}$ is the standard normal density function ϕ.

The optimal allocation of items to examinees aims to maximizing a functional of the information matrix M(h). One can justify and use different such functionals called optimality criteria (see e.g. Atkinsson et al., 2007). A popular criterion with good properties is the D‐optimality criterion, which maximizes the determinant of the information matrix, $\det M(h)=\prod_{i=1,\text{…},n}\det M_{i}(h_{i})$. We will use D‐optimality in this paper, but our method can easily be applied to other optimality criteria.

As described earlier, the design or allocation rule for item i is a function $h_{i}$ with $0\leq h_{i}(\theta )\leq h_{tot}(\theta )$. However, when there is no pair of items which have exactly the same item parameter vectors, Ul Hassan and Miller (2021) showed that a D‐optimal design allocates an item either completely or not at all ($h_{i}(\theta )\in \{0,h_{tot}(\theta )\}$) to all examinees having abilities in intervals. For the numerical computation of the optimal design, we follow Ul Hassan and Miller (2021) and discretize Θ by dividing it into small intervals $[\theta_{j},\theta_{j+1}],j=1,\text{…},J,$ of length e.g. .01 or .001 and assume that either $h_{i}(\theta )=h_{tot}(\theta )$ or $h_{i}(\theta )=0$ on each such interval. As the intervals are chosen with such small length, the discretization is practically not relevant.

This theory developed for optimizing item calibration designs builds on traditional optimal design theory for logistic regression; see Chapter 22 of Atkinsson et al. (2007). Both in traditional optimal design theory and in the variant considered here, the optimal design depends on the unknown parameters ζ because we consider a model non‐linear in the parameters. One way to deal with that is to compute the optimal design based on best guess values for the parameters. We say then that the derived design is locally optimal for the best guess parameter values. When using this method, the best guess values can be based on either expert judgement or results from a smaller precalibration study (Ul Hassan & Miller, 2024). Miller and Fackle‐Fornius (2024) discuss a real calibration study where each item was precalibrated by around 200 examinees before any item parameter estimates were available. Consequences from misspecification of these best guess values were investigated for examples of single items (Ul Hassan & Miller, 2019, Supporting Information) and for the more challenging situation of multidimensional items (Ul Hassan & Miller, 2024) where misspecifications of realistic size were studied. A conclusion in these references is that the optimal design is usually still better than an RD as long as the misspecification is not too severe. In a simulation study with best guess values based on precalibration with 200 examinees, Bjermo et al. (2024) see indications of a non‐severe efficiency loss of the optimal design.

### 3.2 Information matrix adjusted for uncertainty in abilities

We will further develop the optimal calibration design method described in Section 3.1 to account for the fact that the examinees' abilities θ are unknown when allocating items according to the optimal design. We have estimated abilities $\hat{\theta }$ available from the operational test, and their uncertainty can be quantified by $g(\theta |\hat{\theta })$. The function g could be viewed as a prior on θ before the calibration design is determined. The prior can then be replaced by a posterior based on information and/or results from the operational items. The probability in Equation (1) can now be averaged over the prior information $g(\theta |\hat{\theta })$. For an examinee with estimated ability $\hat{\theta }$, the probability to correctly respond to calibration item i is

(2) $${\tilde{p}}_{i}(\hat{\theta })=\int_{\Theta }p_{i}(\theta )g(\theta |\hat{\theta })d\theta .$$

We would like to find the standardized information matrix $M(h)=\text{diag}(M_{1}(h_{1}),...,M_{n}(h_{n}))$ with

(3) $$M_{i}(h_{i})=\int_{\Theta }{\tilde{p}}_{i}(\hat{\theta })(1-{\tilde{p}}_{i}(\hat{\theta }))\left(\frac{\partial \tilde{\eta }(\hat{\theta })}{\partial \zeta_{i}}\right){\left(\frac{\partial \tilde{\eta }(\hat{\theta })}{\partial \zeta_{i}}\right)}^{T}h_{i}(\hat{\theta })d\hat{\theta },$$

where ${\tilde{\eta }}_{i}(\hat{\theta })=\log\left(\frac{{\tilde{p}}_{i}(\hat{\theta })}{1-{\tilde{p}}_{i}(\hat{\theta })}\right)$.

As mentioned before, it is sufficient to consider designs that allocate an item either completely or not at all to all examinees having abilities in intervals. To compute the information matrix for a design, we therefore need the partial information matrices

$$M_{ij}=\int_{{\hat{\theta }}_{j}}^{{\hat{\theta }}_{j+1}}{\tilde{p}}_{i}(\hat{\theta })(1-{\tilde{p}}_{i}(\hat{\theta }))\left(\frac{\partial \tilde{\eta }(\hat{\theta })}{\partial \zeta_{i}}\right){\left(\frac{\partial \tilde{\eta }(\hat{\theta })}{\partial \zeta_{i}}\right)}^{T}h_{tot}(\hat{\theta })d\hat{\theta }.$$

The information matrix for a given design can then be computed as a sum of partial information matrices $M_{ij}$. The next step is to find the derivative $\frac{\partial {\tilde{\eta }}_{i}(\hat{\theta })}{\partial \zeta_{i}}$. As ${\tilde{\eta }}_{i}(\hat{\theta })=\log\left(\frac{{\tilde{p}}_{i}(\hat{\theta })}{1-{\tilde{p}}_{i}(\hat{\theta })}\right)$, the derivative is

(4) $$\frac{\partial {\tilde{\eta }}_{i}(\hat{\theta })}{\partial \zeta_{i}}=\frac{1-{\tilde{p}}_{i}(\hat{\theta })}{{\tilde{p}}_{i}(\hat{\theta })}\frac{{\tilde{p}}_{i}^{\prime }(\hat{\theta })-{\tilde{p}}_{i}^{\prime }(\hat{\theta }){\tilde{p}}_{i}(\hat{\theta })+{\tilde{p}}_{i}^{\prime }(\hat{\theta }){\tilde{p}}_{i}(\hat{\theta })}{{\left(1-{\tilde{p}}_{i}(\hat{\theta })\right)}^{2}}=\frac{{\tilde{p}}_{i}^{\prime }(\hat{\theta })}{{\tilde{p}}_{i}(\hat{\theta })(1-{\tilde{p}}_{i}(\hat{\theta }))},$$

where

$$\begin{array}{lll} {\tilde{p}}_{i}^{\prime }(\hat{\theta }) & = & \frac{\partial {\tilde{p}}_{i}(\theta )}{\partial \zeta_{i}}=\frac{\partial }{\partial \zeta_{i}}\int_{\Theta }p_{i}(\theta )g(\theta |\hat{\theta })d\theta =\int_{\Theta }\frac{\partial }{\partial \zeta_{i}}p_{i}(\theta )g(\theta |\hat{\theta })d\theta \\ & = & \int_{\Theta }\frac{\partial p_{i}(\theta )}{\partial \eta_{i}(\theta )}\frac{\partial \eta_{i}(\theta )}{\partial \zeta_{i}}g(\theta |\hat{\theta })d\theta . \end{array}$$

Assuming a 2PL model, it means that

$$\frac{\partial {\tilde{p}}_{i}(\theta )}{\partial a_{i}}=\int_{\Theta }(\theta -b_{i})p_{i}(\theta )(1-p_{i}(\theta ))g(\theta |\hat{\theta })d\theta ,$$

and

$$\frac{\partial {\tilde{p}}_{i}(\theta )}{\partial b_{i}}=\int_{\Theta }-a_{i}p_{i}(\theta )(1-p_{i}(\theta ))g(\theta |\hat{\theta })d\theta .$$

Given the probability ${\tilde{p}}_{i}(\hat{\theta })$ and the derivatives, the information matrix is

(5) $$\begin{array}{lll} M_{i}(h_{i}) & = & \int_{\Theta }{\tilde{p}}_{i}(\hat{\theta })(1-{\tilde{p}}_{i}(\hat{\theta }))\left(\frac{\partial \tilde{\eta }(\hat{\theta })}{\partial \zeta_{i}}\right){\left(\frac{\partial \tilde{\eta }(\hat{\theta })}{\partial \zeta_{i}}\right)}^{T}h_{i}(\hat{\theta })d\hat{\theta }\\ & = & \int_{\Theta }\frac{1}{{\tilde{p}}_{i}(\hat{\theta })(1-{\tilde{p}}_{i}(\hat{\theta }))}\Psi (\hat{\theta })\Psi {\left(\hat{\theta }\right)}^{T}h_{i}(\hat{\theta })d\hat{\theta }\\ & = & \overset{J}{\underset{j=1}{\sum }}\int_{\theta_{j}}^{\theta_{j+1}}\frac{1}{{\tilde{p}}_{i}(\hat{\theta })(1-{\tilde{p}}_{i}(\hat{\theta }))}\left(\begin{array}{cc} \psi_{11}(\hat{\theta }) & \psi_{12}(\hat{\theta })\\ \psi_{21}(\hat{\theta }) & \psi_{22}(\hat{\theta }) \end{array}\right)h_{i}(\hat{\theta })d\hat{\theta }\\ & \approx & \overset{J}{\underset{j=1}{\sum }}\frac{1}{{\tilde{p}}_{i}(t_{j})(1-{\tilde{p}}_{i}(t_{j}))}\left(\begin{array}{cc} \psi_{11}(t_{j}) & \psi_{12}(t_{j})\\ \psi_{21}(t_{j}) & \psi_{22}(t_{j}) \end{array}\right)h_{i}(t_{j})(\theta_{j+1}-\theta_{j}), \end{array}$$

where

(6) $$\Psi (\hat{\theta })=\int_{\Theta }\left(\begin{array}{c} \left(\theta -b_{i}\right)\\ -a_{i} \end{array}\right)p_{i}(\theta )(1-p_{i}(\theta ))g(\theta |\hat{\theta })d\theta ,$$

(7) $$\psi_{11}(\hat{\theta })={\left(\int_{\Theta }(\theta -b_{i})p_{i}(\theta )(1-p_{i}(\theta ))g(\theta |\hat{\theta })d\theta \right)}^{2},$$

(8)

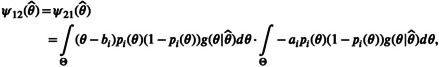

(9) $$\psi_{22}(\hat{\theta })={\left(\int_{\Theta }-a_{i}p_{i}(\theta )(1-p_{i}(\theta ))g(\theta |\hat{\theta })d\theta \right)}^{2},$$

and $t_{j}=(\theta_{j}+\theta_{j+1})/2$. The approximation of the integral in (5) corresponds to the Riemann integration rule, see e.g., Givens and Hoeting (2013), Chapter 5.

These results make it possible to adapt the optimal design method acknowledging uncertainty in the examinees' abilities. It remains to quantify the distribution $g(\theta |\hat{\theta })$. Two strategies for determining this distribution are described in the following section.

### 3.3 Determining the distribution $g(\theta |\hat{\theta })$

#### 3.3.1 Approximating $g(\theta |\hat{\theta })$ with a normal distribution

Let ${\hat{\theta }}_{m}$ be the MLE of an examinee's ability based on m operational items. Also, let ${\hat{\sigma }}_{m}^{2}$ be the estimated variance of ${\hat{\theta }}_{m}$ which is asymptotically equal to $I{\left(\theta_{m}\right)}^{-1}$ (Lord, 1983), where $I(\theta )=\sum_{i=1}^{m}I_{i}(\theta )$ is the test information, and

(10) $$I_{i}(\theta )=a_{i}^{2}p_{i}(\theta )(1-p_{i}(\theta )).$$

is the item information (Baker & Kim, 2004, 2017) for the 2PL IRT model.

It has been proven (Chang, 1996; Chang & Stout, 1993) that under some regularity conditions on the item response function and prior distribution, the posterior distribution $\frac{\left({\hat{\theta }}_{m}-\theta \right)}{{\hat{\sigma }}_{m}}$ converges in probability and almost surely to the standard normal distribution as the number of test items m tends to infinity.

We can use this result and let the distribution of $g(\theta |\hat{\theta })$ be the posterior of θ, which can be approximated as $N({\hat{\theta }}_{m},{\hat{\sigma }}_{m}^{2})$ for large enough m. We consider the number of operational items fixed and will drop the index m from $\hat{\theta }$. We therefore let $g(\theta |\hat{\theta })=N(\hat{\theta },I^{-1}(\hat{\theta }))$ in the preceding expressions for the information matrix, where $\hat{\theta }$ is estimated by items with parameter vectors $a={\left(a_{1},\text{…},a_{m}\right)}^{T}$ and $b={\left(b_{1},\text{…},b_{m}\right)}^{T}$ of the operational test which are assumed to be known. Usually, the items are stored in an item bank where the item parameters are estimated with enough precision to be treated as the true ones (see, e.g. van der Linden & Pashley, 2010, p. 6). The investigation of He and Chen (2020) suggests that 600 examinees who have calibrated the operational items give a root MSE less than .2 for the a‐parameter, and less than .15 for the b‐parameter for most of the compared methods.

When assuming $g(\theta |\hat{\theta })=N(\hat{\theta },I^{-1}(\hat{\theta }))$, the standardized information matrix can be calculated for different values of $\hat{\theta }$.

This means that the design in this case can be derived before any examinee has taken the operational items.

A problem that could arise, especially in CATs, is that the MLE is biased in case of perfect/near‐perfect or zero/near‐zero score to the operational items (Lord, 1983). This suggests using, e.g.  the Bayesian modal or maximum a posteriori (MAP) estimate or the expected a posteriori (EAP) instead; see Baker and Kim (2004) for a description.

#### 3.3.2 Replacing $g(\theta |\hat{\theta })$ with posterior draws

If the number of operational items is not sufficient to approximate $g(\theta |\hat{\theta })$ with the normal distribution, we can still proceed by approximating the integrals in (2) and ((6), (7), (8), (9)) as

(11) $${\tilde{p}}_{i}(\theta )\approx S^{-1}\overset{S}{\underset{s=1}{\sum }}p_{i}(\theta^{\left(s\right)}),$$

and, e.g.,

(12) $$\psi_{11}(\theta )\approx {\left(S^{-1}\overset{S}{\underset{s=1}{\sum }}(\theta^{\left(s\right)}-b_{i})p_{i}(\theta^{\left(s\right)})(1-p_{i}(\theta^{\left(s\right)}))\right)}^{2},$$

where the vector $\theta =(\begin{array}{c} \theta^{\left(1\right)},\text{…},\theta^{\left(S\right)} \end{array})$ consists of the posterior distribution draws for an examinee's ability based on the results of the operational test; see van der Linden and Ren (2015) who apply similar integral approximations. This is equivalent to the Monte Carlo approximation of an integral where $\theta^{\left(1\right)},\text{…},\theta^{\left(S\right)}$ are draws from the probability density. See Robert and Casella (2010) for a description of Monte Carlo integration.

When no assumption is made about the distribution of $g(\theta |\hat{\theta })$, the estimates of the ability ${\hat{\theta }}_{j}$ (e.g. EAP‐estimates) and the ability posterior draws $\theta_{j}$ for examinee j are available first after the operational items have been taken. The standardized information matrix can now be approximated as

(13) $$M_{i}(h_{i})\approx \overset{N}{\underset{j=1}{\sum }}\frac{1}{{\tilde{p}}_{i}(\theta_{j})(1-{\tilde{p}}_{i}(\theta_{j}))}\left(\begin{array}{c} \psi_{11}(\theta_{j})\psi_{12}(\theta_{j})\\ \psi_{21}(\theta_{j})\psi_{22}(\theta_{j}) \end{array}\right)h_{i}({\tilde{t}}_{j})({\hat{\theta }}_{j+1}-{\hat{\theta }}_{j}),$$

where ${\tilde{p}}_{i}(\theta_{j})$ and $\psi_{11}(\theta_{j})$ are calculated using (11) and (12); and $\psi_{12}(\theta_{j})=\psi_{21}(\theta_{j})$, $\psi_{22}(\theta_{j})$ are calculated similarly. Here, N is the number of examinees taking the operational test. The estimates ${\hat{\theta }}_{j}$, j=1,…N, are sorted and ${\tilde{t}}_{j}$ are calculated as ${\tilde{t}}_{j}=({\hat{\theta }}_{j}+{\hat{\theta }}_{j+1})/2$.

The estimates ${\hat{\theta }}_{j}$, j=1,…N, of the examinee's abilities are assumed to follow the standard normal distribution, so $h_{i}(t_{j})$ is calculated as the density of the standard normal distribution. Note that this is the distribution of the population of all examinees' ability estimates, whereas $g(\theta |\hat{\theta })$ quantifies the uncertainty of the ability of a single examinee.

When we cannot approximate $g(\theta |\hat{\theta })$ with the normal distribution, the ability estimates and posterior draws for every examinee are based on the operational items. This means that the operational items must be given and analysed for all examinees before determining the optimal design, making it unsuitable for an integrated set‐up when calibrating items.

### 3.4 Relative efficiency

To compare different designs (item allocation rules), one can consider relative efficiencies of one design $h^{\left(1\right)}$ versus another design $h^{\left(2\right)}$. For the D‐criterion, the relative D‐efficiency is the ratio of the determinants up to the power of 1/p, where p is the number of model parameters; see Berger and Wong (2009) and Ul Hassan and Miller (2019):

$$RE_{D}={\left[\det\left\{M\left(h^{\left(1\right)}\right)\right\}/\det\left\{M\left(h^{\left(2\right)}\right)\right\}\right]}^{1/p}.$$

A relative efficiency of $h^{\left(1\right)}$ versus $h^{\left(2\right)}$ smaller than 1 means that design $h^{\left(2\right)}$ is better. The relative efficiency can be interpreted in terms of sample size: The design $h^{\left(2\right)}$ needs $RE_{D}$ times as many examinees as $h^{\left(1\right)}$ to have the same precision of estimates (measured with the D‐criterion).

Focusing on a specific item i, we consider its information matrix $M_{i}(h_{i})$. The relative D‐efficiency for this item of $h_{i}^{\left(1\right)}$ versus $h_{i}^{\left(2\right)}$ is ${\left[\det\left\{M_{i}\left(h_{i}^{\left(1\right)}\right)\right\}/\det\left\{M_{i}\left(h_{i}^{\left(2\right)}\right)\right\}\right]}^{1/2}$, such that $RE_{D}$ is the geometric mean of the item efficiencies. If we are interested in the precision of a single parameter estimate, an approximation of its variance is proportional to $c^{T}M_{i}{\left(h_{i}\right)}^{-1}c$ with $c={\left(1,0\right)}^{T}$ for the first parameter (discrimination, $a_{i}$) and $c={\left(0,1\right)}^{T}$ for the second parameter (difficulty, $b_{i}$). The proportionality constant depends on the sample size. Comparing two designs $h_{i}^{\left(1\right)}$ and $h_{i}^{\left(2\right)}$, the ratio of the two variance approximations

$$c^{T}M_{i}{\left(h_{i}^{\left(2\right)}\right)}^{-1}c/c^{T}M_{i}{\left(h_{i}^{\left(1\right)}\right)}^{-1}c,$$

is the parameter efficiency of $h_{i}^{\left(1\right)}$ versus $h_{i}^{\left(2\right)}$ which is called c‐efficiency (Atkinsson et al., 2007, section 21).

### 3.5 Extensions to other models

In the previous sections, we introduced the methodology for the 2PL model, but the theory for handling the uncertainty in abilities is not limited to this model. Here, we briefly discuss how the theory can be extended and applied to other models. Operational items may be modelled using other dichotomous or polytomous IRT models, whereas the 2PL model might still be used for the calibration items. Alternatively, the models for the calibration items might be different from the 2PL model.

#### 3.5.1 Other models for the operational items

When the operational items are modelled including other models, we will adjust the item information (10) accordingly. For example, for the three‐parameter logistic (3PL) model with parameters $\zeta_{i}=(a_{i},b_{i},c_{i})$ and

(14) $$p_{i}(\theta )=P(Y_{i}=1|\theta ,a_{i},b_{i},c_{i})=c_{i}+\frac{1-c_{i}}{1+e^{-a_{i}(\theta -b_{i})}},$$

the item information is $I_{i}(\theta )=a_{i}^{2}(1-p_{i}(\theta ))\frac{{\left((p_{i}(\theta )-c_{i})\right)}^{2}}{p_{i}(\theta ){\left(1-c_{i}\right)}^{2}}$ (Baker & Kim, 2017).

When $g(\theta |\hat{\theta })$ cannot be approximated with the normal distribution, we use the posterior draws for the examinees' abilities based on the operational items that can belong to other models than the 2PL model. The posterior draws from the operational test is then used to approximate the integrals. It is, therefore, not necessary for the calibration items to belong to the same model as the operational items.

#### 3.5.2 Other models for the calibration items

If other IRT models than the 2PL are used for some item, formula (5) is still valid for the information matrix. The vector $\Psi (\hat{\theta })$ needs to be adjusted based on the model. For the three‐parameter logistic (3PL) model according to (14), the information matrix will follow formula (5) with

$$\Psi (\hat{\theta })=\int_{\Theta }\left(\begin{array}{c} \frac{\theta -b_{i}}{1-c_{i}}(p_{i}(\theta )-c_{i})\\ -\frac{a_{i}}{1-c_{i}}(p_{i}(\theta )-c_{i})\\ \frac{1}{1-c_{i}} \end{array}\right)(1-p_{i}(\theta ))g(\theta |\hat{\theta })d\theta .$$

This means that

$$M_{i}(h_{i})=\int_{\Theta }\frac{1}{{\tilde{p}}_{i}(\hat{\theta })(1-{\tilde{p}}_{i}(\hat{\theta }))}\left(\begin{array}{ccc} \psi_{11}(\hat{\theta }) & \psi_{12}(\hat{\theta }) & \psi_{13}(\hat{\theta })\\ \psi_{21}(\hat{\theta }) & \psi_{22}(\hat{\theta }) & \psi_{23}(\hat{\theta })\\ \psi_{31}(\hat{\theta }) & \psi_{32}(\hat{\theta }) & \psi_{33}(\hat{\theta }) \end{array}\right)h_{i}(\hat{\theta })d\hat{\theta },$$

where 

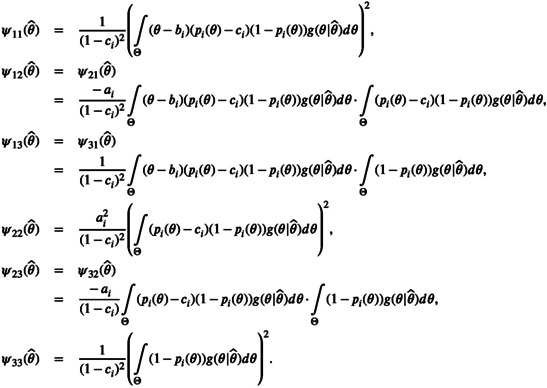




## 4 EVALUATION OF THE UNCERTAINTY‐ADJUSTED DESIGN METHODOLOGY

In this section, we describe the methods we use to evaluate different aspects of the proposed design methodology. We begin in Section 4.1 with the comparison of the information matrices resulting from the two methods of determining $g(\theta |\hat{\theta })$: approximation of a normal distribution or replacing it with ability posterior draws. Then, we specify the configuration of item blocks for the uncertainty‐adjusted optimal designs and their efficiency evaluations. Next, we describe the set‐up of a simulation study to evaluate and compare item parameter recoveries under different designs. Section 4.5 illustrates the implementation of the R‐package optical. The results of all of these evaluations are presented in Section 5.

### 4.1 Comparing information matrices for the two methods to determine $g(\theta |\hat{\theta })$

To evaluate the difference in information between the two methods, we will compare the values of the standardized information matrices. The calculation of the full standardized information matrix is made by using the approximation in (5). The approximation includes integrals that also need to be calculated, e.g., by some approximation for values of $t_{j}$.

We compare the standardized information matrices $M_{i}(h_{i})$ resulting from two methods introduced in Section 3.3 and outlined earlier. We will calculate $M(h_{i})$ for two items i=1,2, where $h_{i}(\theta )=h_{tot}(\theta )∼N(0,1)$. That is, $M_{i}(h_{i})$ is calculated for the whole span of θ for the items just for comparison. We generate a response matrix by using 40 true item parameters (a,b) estimated from SweSAT data (Umeå University, 2022) assuming a 2PL model. N=1000 examinee abilities θ are randomly selected from N(0,1). The generated response matrix represents item responses to the operational test. We use the R‐package mirt() (Chalmers, 2023) to estimate the EAP estimates of the abilities and to produce Metropolis Hastings samples from the posterior for each of the 1000 examinees.

Without approximating $g(\theta |\hat{\theta )}$ with the normal distribution, we first use the Monte Carlo approximation using the S ability posterior samples for every examinee and then calculate the standardized information matrix by approximating the sum as described in Equation (13).

When assuming $g(\theta |\hat{\theta })=N(\hat{\theta },I^{-1}(\hat{\theta }))$, we first calculate the integrals (2) and ((6), (7), (8), (9)) and then $M_{i}(h_{i})$ is calculated as in (5) with a step size of .01.

We calculate $M_{i}(h_{i})$ for two calibration items with parameters $a_{1}=1,b_{1}=-1$ and $a_{2}=1.5,b_{2}=1$ by using the two methods, where $M_{i}(h_{i})$ using the Monte Carlo approximation is calculated for S=10,000 posterior draws.

### 4.2 Uncertainty‐adjusted optimal designs

Our proposed optimal design method that adjusts the optimal design to account for the uncertainty in abilities depends on the amount of information available through the operational test. The number of operational test items (m), as well as the operational test item parameters, influences the amount of information obtained about the examinees' abilities. In Section 5.2, we examine how the results depend on the ability information available. It is obvious that the amount of information about the examinees' ability is increasing with an increasing number of operational test items m, and we vary therefore this important influence factor as m=6,12,18,30,60,120. We also include the case of known abilities, corresponding to m=∞. Other factors influencing the ability information are the parameters of the items in the operational test. We choose the item parameters in a way that they do not interfere when we investigate increasing m and that they are comparable over the different m. To keep it simple, we therefore use specifically operational items with difficulties that are equidistant between −1.5 and 1.5. We keep the discrimination of the items initially constant and assume that all operational items have equal discrimination $a_{op}=1$. Of course, no real operational test will have items all having the same discrimination, but our setting represents a test of size m with difficulties spread and abilities around $a_{op}$.

Regarding the items to be calibrated, we investigate three cases where a block of two, three or four items should be calibrated, respectively. In Table 1, the assumed item parameter values for the three block sizes are specified. These values are selected to illustrate how the design properties and efficiencies are affected by a variation in difficulty and discrimination parameter values. In all cases, we assume that the test takers have standard normally distributed abilities, that is, $h_{tot}$ has a N(0,1)‐density.

**TABLE 1:** Assumed true calibration item parameters for the three block size examples in Section 4.2.

Example item	Two‐item block		Three‐item block			Four‐item block			
	1	2	1	2	3	1	2	3	4
a	1.6	1.6	1	2	2.5	1.5	1	1	1.5
b	−1	1	−1.5	.5	2	−1.5	−.25	.25	1.5

### 4.3 Efficiency evaluations

The proposed uncertainty‐adjusted D‐optimal design is evaluated in terms of efficiency. The evaluations are made in terms of different types of efficiency, and in comparison to different designs, as described in the following.

#### 4.3.1 Efficiency compared to the RD

We calculate the relative D‐ and the individual c‐efficiencies (Section 3.4) of the uncertainty‐adjusted D‐optimal designs versus an RD. The discrimination parameters of the operational items as well as the number of items m are held fixed.

#### 4.3.2 Efficiency if uncertainty in abilities is ignored

One could ignore the uncertainty in abilities and compute and use a standard D‐optimal design pretending that abilities are known while they are in fact estimated. This leads to an efficiency loss compared to the D‐optimal design where the uncertainty is respected. To quantify the loss, we use the relative D‐efficiency for the uncertainty‐adjusted D‐optimal design versus an RD and compare it to the relative D‐efficiency for the standard D‐optimal design using known abilities versus an RD. We use the three examples described in Section 4.2 and the same values for the discrimination and difficulty parameters of the operational items with the number of items m varied between 6 and 120. We will also compare the relative D‐efficiency for the D‐optimal design accounting for uncertainty and the D‐optimal design assuming known abilities.

To compare the relative individual parameter efficiencies for D‐optimal design using known abilities and accounting for uncertainty, we calculate the c‐efficiencies of the discrimination and difficulty parameters as well as the D‐efficiency per item.

### 4.4 Bias and MSE based on simulations

The methods used in Sections 4.2 and 4.3.2 are based on numerical optimization of the algebraic expressions derived in Section 3. These algebraic expressions are based on asymptotic variances, and the properties of the estimators for finite samples need to be examined through simulations. We investigate bias and MSE of the parameter estimators by a simulation study with N=500 examinees. We conduct 1000 repetitions in this study to report the average bias and average MSE over the 1000 repetitions. The true abilities of the examinees were simulated from the standard normal distribution. Then, we estimate them using the R‐package mirt (Chalmers, 2023) based on m=30 items from an operational test, having difficulties and abilities as described in Section 4.2. The uncertainty‐adjusted D‐optimal design and the D‐optimal design for known abilities were applied by assigning the item based on the estimated ability; furthermore, an RD allocating each item with equal probability was also applied. The response to the new item was then based on the true abilities. The two parameters $a_{i}$ and $b_{i}$ were then estimated with the MLE separately for each item using the estimated abilities as independent variable.

### 4.5 Computing the optimal designs and efficiencies with the R‐package optical

**FIGURE 1:**
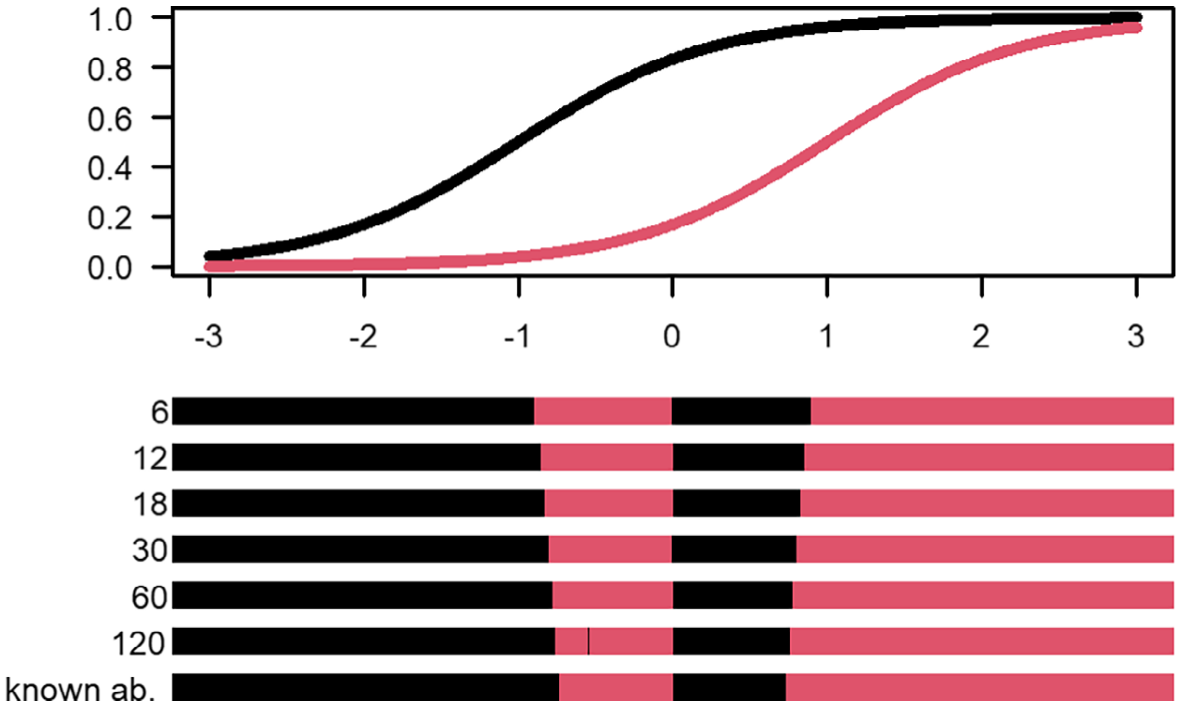
Top panel: Assumed true calibration item characteristic curves for the two‐item block. Bottom panel: Uncertainty‐adjusted D‐optimal designs based on an operational test with m= 6, 12, 18, 30, 60, 120 operational items having discrimination $a_{op}=1$ and difficulty between −1.5 and 1.5, as well as the standard D‐optimal design for known abilities. The black and red intervals determine which of the two items in the block (given in Table 1) an examinee with a certain estimated ability will be assigned to calibrate; the interval colour matches the colour of the item characteristic curves.

The design and efficiency calculations have been derived with the R‐package optical (Ul Hassan & Miller, 2023), available at the CRAN repository (R Core Team, 2024). We first illustrate the computation of the optimal designs in Section 4.2 for the example of m=6 operational items and the block with two calibration items (see Figure 1). The R‐code to calculate the D‐optimal calibration design is:

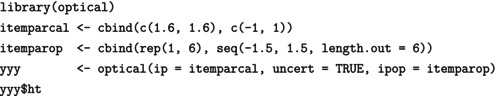

In the function optical, the options ip and ipop define the item parameters of the calibration and operational test, respectively. By setting uncert = TRUE, one obtains the design, taking the ability‐uncertainty after the operational item into account. If one sets uncert = FALSE (default) instead, one can obtain the design for known abilities according to the method described by Ul Hassan and Miller (2019) and Ul Hassan and Miller (2021). The output obtained shows then the ability intervals (for standard normally distributed abilities) together with the item for each interval.

For efficiency calculations, the function efficiency can be used, again with the option uncert = TRUE to apply the method described in this article. This function computes the relative efficiency of a previously computed design versus the RD. For example, the first D‐efficiency value of 1.14 in Table 3 can be computed as follows:

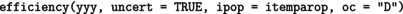

To use the package for computing individual parameter‐efficiencies, we note that the c‐optimality criterion is a special case of the so‐called L‐optimality (Atkinsson et al., 2007) with L‐matrix $L=\text{c}{\text{c}}^{⊤}$, where the vector c is ${\left(1,0\right)}^{⊤}$ for the discrimination parameter and ${\left(0,1\right)}^{⊤}$ for the difficulty parameter. To calculate the first two parameter efficiency values in Table 4, we use the code:

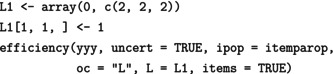

Here, we have first defined the L‐matrices for each item, giving us the parameter efficiency for the discrimination: The first two indices of the array define the L‐matrix for the item number in the third index (we set here the left‐upper entry in the L‐matrices for all items to 1, the other entries remain 0). In the function call, we specify the L‐optimality by oc = "L" and by specifying the L‐matrix. The option items = TRUE gives us an output with the efficiency values. First, for each item separately and in the last column, the overall efficiency. When this option is specified, the output also contains two extra rows with the criterion values for the optimal and the RD, respectively, and their ratio is the efficiency.

## 5 RESULTS

### 5.1 Comparing information matrices

We compare the information matrices when approximating $g(\theta |\hat{\theta })$ with the normal distribution and when using the posterior draws. They are then compared to when the true abilities are assumed to be known.

Table 2 shows that the Monte Carlo approximation with S=10,000 posterior draws and the normal distribution approximation give results that differ with a magnitude of about .01 to .001. When using the normal distribution approximation, the information for the parameters $a_{i}$ and $b_{i}$ in the diagonal of the matrix is, as expected, lower compared to the case of true abilities, but the difference is not too big. It suggests that the information about abilities from 40 operational items from a real situation is enough to justify the normality approximation. The values of the information matrices are very similar when using 1000 or 100 posterior draws compared to 10,000. The time it takes to sample the posterior draws depends linearly on the number of posterior draws. If it takes, e.g., 5 min to sample 10,000 posterior draws for every 1000 examinees, it takes .5 min and 3 s for 1000 and 100 posterior draws, respectively.

**TABLE 2:** Standardized information matrix for items $a_{1}=1,b_{1}=-1$ (row 1) and $a_{2}=1.5,b_{2}=1$ (row 2).

$a_{i}$	$b_{i}$	10,000 posterior draws	Normal distr. approximation	True abilities
1	−1	$\left(\begin{array}{cc} .1918 & -.1188\\ -.1188 & .1732 \end{array}\right)$	$\left(\begin{array}{cc} .2013 & -.1239\\ -.1239 & .1724 \end{array}\right)$	$\left(\begin{array}{cc} .2158 & -.1253\\ -.1253 & .1779 \end{array}\right)$
1.5	1	$\left(\begin{array}{cc} .1094 & .1059\\ .1059 & .2951 \end{array}\right)$	$\left(\begin{array}{cc} .1046 & .1088\\ .1088 & .3066 \end{array}\right)$	$\left(\begin{array}{cc} .1175 & .1123\\ .1123 & .3146 \end{array}\right)$

*Note*: Comparing the Monte Carlo approximation with S=10,000 posterior draws, the normal distribution approximation and using true abilities.

### 5.2 Uncertainty‐adjusted D‐optimal designs

**FIGURE 2:**
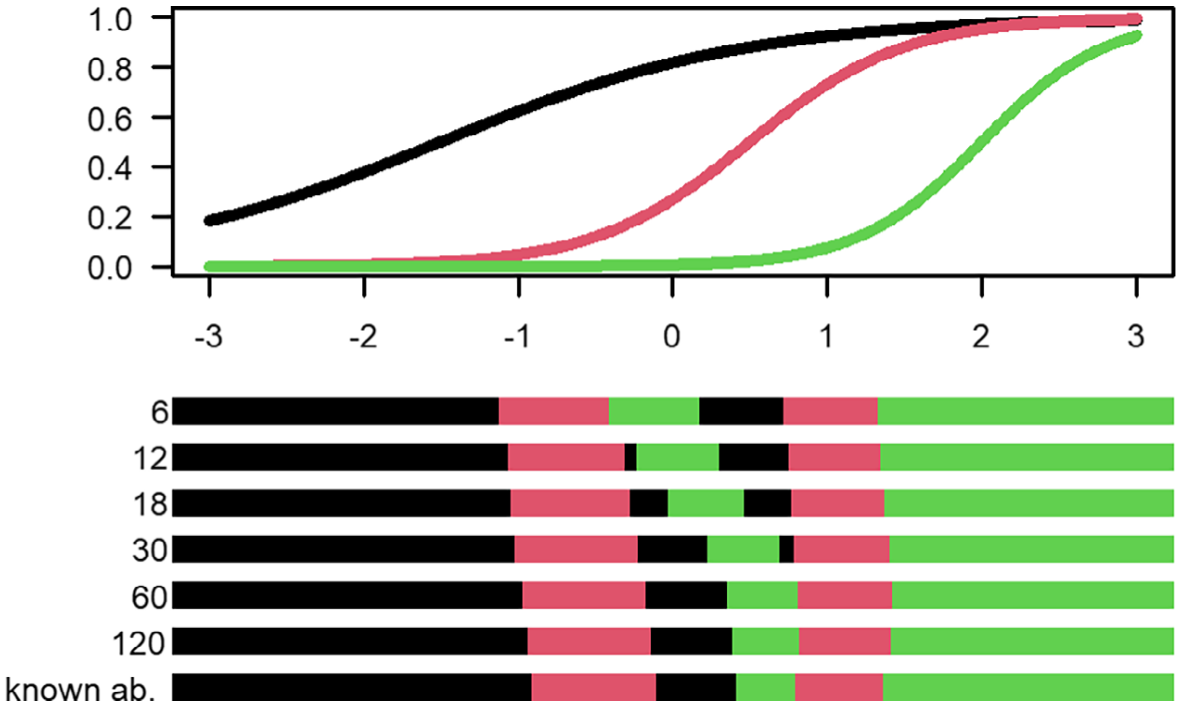
Top panel: Assumed true calibration item characteristic curves for three items in calibration block. Bottom panel: Uncertainty‐adjusted D‐optimal designs with uncertainty in abilities based on an operational test with m= 6, 12, 18, 30, 60, 120 operational items having discrimination $a_{op}=1$ and difficulty between −1.5 and 1.5, as well as the standard D‐optimal design for known abilities. The black, red and green intervals determine which of the three items in the block (given in Table 1) an examinee with a certain estimated ability will be assigned to calibrate; the interval colour matches the colour of the item characteristic curves.

**FIGURE 3:**
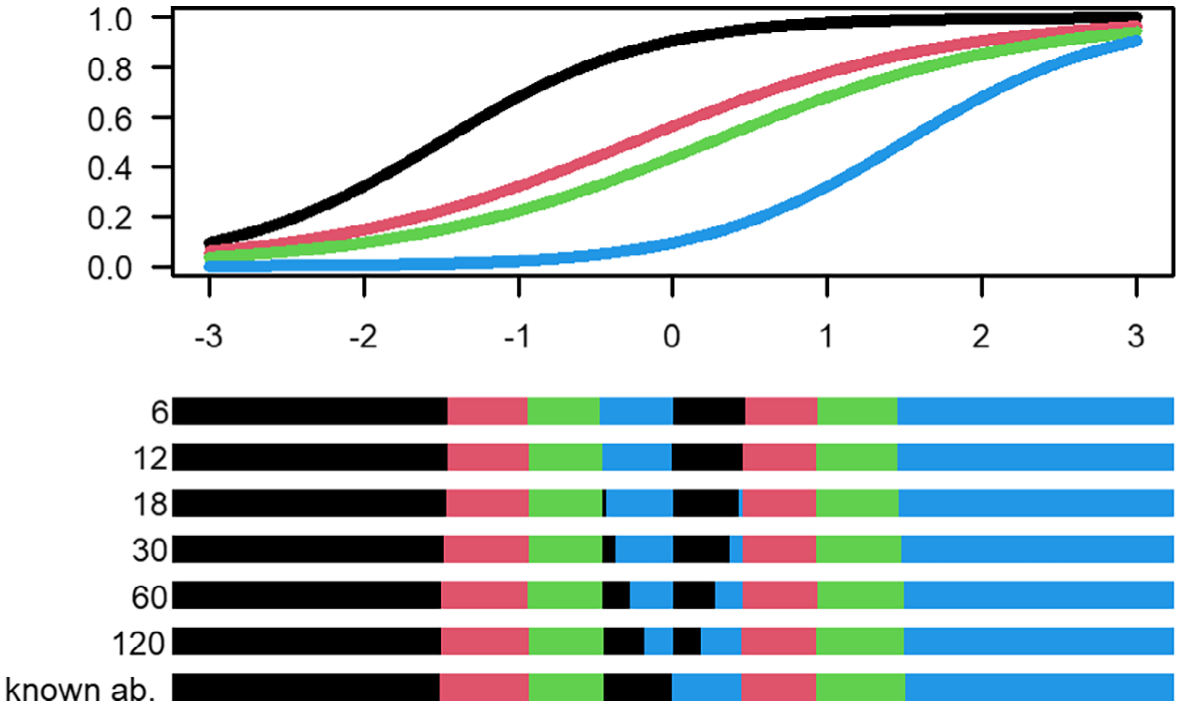
Top panel: Assumed true calibration item characteristic curves for four items in calibration block. Bottom panel: Uncertainty‐adjusted D‐optimal designs with uncertainty in abilities based on an operational test with m= 6, 12, 18, 30, 60, 120 operational items having discrimination $a_{op}=1$ and difficulty between −1.5 and 1.5, as well as the standard D‐optimal design for known abilities. The black, red, green and blue intervals determine which of the four items in the block (given in Table 1) an examinee with a certain estimated ability will be assigned to calibrate; the interval colour matches the colour of the item characteristic curves.

The uncertainty‐adjusted D‐optimal designs are shown in Figures 1, 2, 3 for the two‐item, three‐item and four‐item block examples in Table 1, respectively. The colour of the intervals indicates which item an examinee with an estimated ability within that interval will calibrate. As shown in Figure 1, an examinee with estimated ability in the black interval will be assigned to calibrate item 1, and an examinee with estimated ability in the red interval will be assigned to calibrate item 2.

Figure 1 shows the uncertainty‐adjusted optimal designs for the two‐item block example. We note that with lower m, there are fewer operational items and thus less information about the abilities, resulting in wider middle intervals in the design, compared to the standard design assuming known abilities. The fact that the middle intervals are wider leads to a more robust design in the sense that it includes more ability levels and therefore allows for some misspecification of the abilities around pivotal points.

Figure 2 shows the uncertainty‐adjusted optimal designs for the three‐item block example. We see here that the two middle intervals for Item 1 (black coloured) and Item 3 (green coloured) change their order starting from m=6 and increasing until the case of known abilities. When considering uncertainty, the distance between the two black Item 1‐intervals is larger compared to the case of known abilities. The same is true for the two green Item 3‐intervals. Again, this achieves a robustness in that the intervals for each item cover a larger range of abilities in the middle regions.

Figure 3 shows the uncertainty‐adjusted optimal designs for the four‐item block example. Similarly as for the previous example, we see that the two middle intervals for Item 1 (black coloured) and Item 4 (blue coloured) change their order, leading to the same type of robustness as for the other block sizes.

### 5.3 Relative D and c‐efficiency

Table 3 shows the relative D‐efficiencies of the uncertainty‐adjusted D‐optimal designs versus an RD. We see that the designs for cases with higher uncertainty/fewer operational test items (lower m) have smaller efficiency, which can be viewed as a price for their increased robustness. Note that the efficiency versus the RD is approaching 1 when the amount of ability‐information from the operational test items becomes smaller. This is reasonable because without information about the abilities, we cannot improve the RD anymore. We see in the results that the efficiencies for the three‐item block are larger than for the other blocks, irrespective of the amount of ability information available (m). The reason is that the precision of very difficult or very easy items (|b| large) can be considerably improved if the right examinees are oversampled to such items. In the three‐item block, the difficult third item with $b_{3}=2$ gains the most from the optimal design. For example, if we take m=∞, the individual item efficiencies for the three‐item block are 1.40, 1.34 and 1.60, respectively, whereas for the four‐item block, they are 1.40, 1.14, 1.14 and 1.40, respectively.

**TABLE 3:** Relative D‐efficiencies of the uncertainty‐adjusted designs compared to a random allocation of items; m=∞ stands for the case of known abilities.

Example	Number of operational items m						
	6	12	18	30	60	120	∞
Two‐item block	1.14	1.17	1.19	1.20	1.22	1.22	1.23
Three‐item block	1.24	1.28	1.31	1.35	1.39	1.41	1.44
Four‐item block	1.15	1.18	1.20	1.22	1.24	1.25	1.26

In these examples, we fixed the discrimination parameter of the operational items and varied the number of items m. Nevertheless, if we fix the number of items and vary the discrimination parameter of the operational items, we see a similar picture. This follows from the formulas for test and item information, $I(\theta )=\sum_{i=1}^{m}I_{i}(\theta )$ and (10), where we see that the test information increases linearly in m and quadratically in a for the simplified case when all $a_{i}=a$ are equal: The information about the examinees' abilities, which we obtain from m operational items with discrimination $a_{op}$, depends on $m\cdot a_{op}^{2}$; for example, if the operational items have only half of the discrimination, we need four times as many of them to obtain approximately the same information about the ability of the examinees. Therefore, we will obtain similar results as above, if we fix m=30 and consider for all operational items $a_{op}=\sqrt{6/30},\sqrt{12/30},\sqrt{18/30},1,\sqrt{2},2$, respectively.

The individual parameter c‐efficiencies; that is, the ratios of the parameter's precision between the optimal and RD, are shown in Table 4. We see that the uncertainty‐adjusted D‐optimal design is better for all parameters and uncertainty levels m, except for the difficulty parameter of the two middle items in the four‐item block for m=6. As was the case for the D‐efficiency, for most of the c‐efficiencies, the efficiency gain of the optimal design is lower with higher uncertainty (lower m). As we optimize the D‐criterion for all items in a block, individual item efficiencies can in a few cases also decrease with increasing m. For example, in the three‐item block example, the uncertainty‐adjusted optimal design for lower m puts more emphasis on the discrimination parameter such that the item efficiencies for a are decreasing while they are strongly increasing for b with m increasing.

**TABLE 4:** Relative c‐efficiency for discrimination $a_{i}$ and difficulty $b_{i}$ of the uncertainty‐adjusted optimal design versus an RD for m∈{6,12,18,30,60,120} and known abilities (m=∞), $a_{op}=1$ and the item block examples in Section 4.2.

m	Optimality	Two‐item block		Three‐item block			Four‐item block			
		Item		Item			Item			
		1	2	1	2	3	1	2	3	4
6	a	1.28	1.28	1.45	1.35	1.60	1.48	1.16	1.16	1.48
	b	1.24	1.24	1.65	1.04	1.68	1.43	.99	.99	1.43
12	a	1.32	1.32	1.43	1.40	1.61	1.62	1.17	1.17	1.62
	b	1.30	1.30	1.86	1.05	1.91	1.54	1.02	1.02	1.54
18	a	1.34	1.34	1.34	1.45	1.53	1.65	1.18	1.18	1.65
	b	1.34	1.34	1.96	1.06	2.02	1.61	1.03	1.03	1.61
30	a	1.35	1.35	1.26	1.52	1.43	1.62	1.21	1.21	1.62
	b	1.38	1.38	2.02	1.09	2.13	1.70	1.03	1.03	1.70
60	a	1.35	1.35	1.28	1.56	1.42	1.57	1.23	1.23	1.57
	b	1.42	1.42	2.09	1.09	2.29	1.79	1.04	1.04	1.79
120	a	1.35	1.35	1.33	1.56	1.42	1.54	1.24	1.24	1.54
	b	1.45	1.45	2.15	1.09	2.43	1.84	1.05	1.05	1.84
∞	a	1.34	1.34	1.36	1.55	1.39	1.53	1.25	1.25	1.53
	b	1.48	1.48	2.21	1.11	2.60	1.89	1.05	1.05	1.89

### 5.4 Ignoring ability uncertainty

**FIGURE 4:**
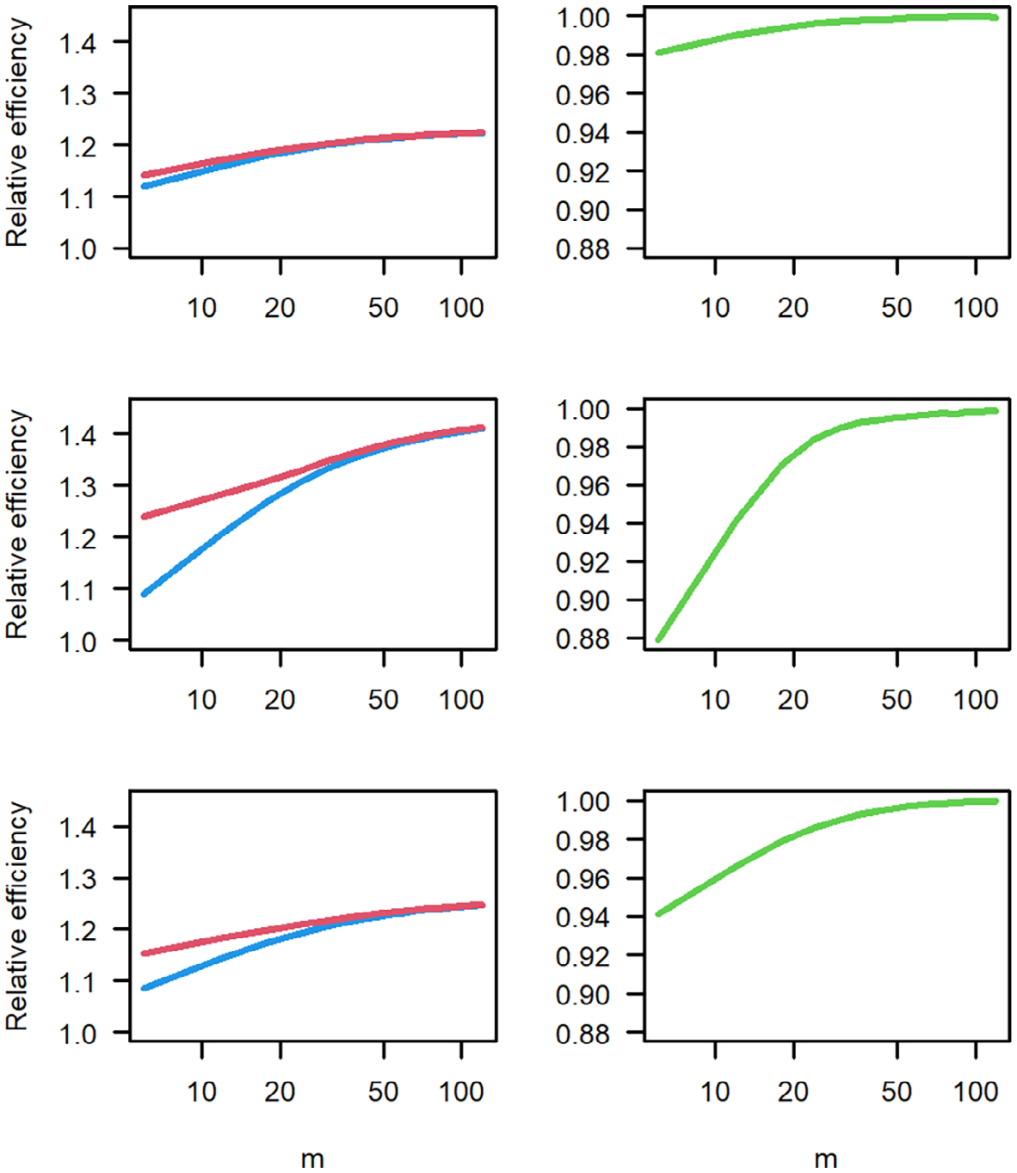
Left panels: Relative D‐efficiency versus random design of the uncertainty‐adjusted D‐optimal design (upper red curve) and the D‐optimal design derived for known abilities (lower blue curve). Right panels: Relative D‐efficiency of D‐optimal design derived for known abilities versus the uncertainty‐adjusted D‐optimal design. Top panel: two‐item block example; middle panel: three‐item block example; lower panel: four‐item block example. In all examples, discrimination of all operational items $a_{op}=1$. Number of operational items m between 6 and 120.

In Figure 4, we compare the two designs for the three examples from Section 4.2 with m between 6 and 120, and $a_{op}=1$. In the left panels, we have the relative D‐efficiencies versus the RD of the two designs. The right panels show the ratio of these values, which is the relative D‐efficiency of the design ignoring the uncertainty in ability versus the uncertainty‐adjusted D‐optimal design.

For the two‐item block, the optimal design calculated pretending that the abilities are known has a high relative efficiency of at least .98 versus the uncertainty‐adjusted optimal design. In the second, three‐item block example, this efficiency can decrease to .88 when there is item information from six 2‐PL items with discrimination $a_{op}=1$. In the third case (four‐item block), the relative efficiency is at least .95.

Therefore, in some cases for smaller number of operational items m, the optimal design calculated ignoring the uncertainty is still good, whereas in other cases, the proper handling of the uncertainty is important. When the number of operational items is larger, the handling of the uncertainty is less important. Note that we used a discrimination of $a_{op}=1$ for these results and that they depend on $m\cdot a_{op}^{2}$. That is, if we have lower discriminating items, a higher number of operational items are necessary to reach the point where the uncertainty can be ignored. However, even if we here identified cases where the appropriate handling of uncertainties is less important, it has to be pointed out that the method proposed here is never worse for a given setting of operational items in terms of D‐efficiency. The only disadvantage of handling the uncertainty in abilities is that the method becomes more complex and that the computing time of the optimal design increases.

Table 5 shows the relative individual parameter c‐efficiencies for estimation of the discrimination (a‐) and the difficulty (b‐) parameter as well as the D‐efficiency per item when we compare the D‐optimal design derived for known abilities versus the uncertainty‐adjusted D‐optimal design. We focus here on the values m∈{6,30} only because these values are enough to understand the behaviour. When the relative efficiency is below 1, this parameter/item is better estimated when handling the uncertainty compared to when ignoring it. We see that handling of the uncertainty is especially important for the estimation of the a‐parameter and especially when we have quite difficult or easy items: most of these efficiencies are smaller than 1, showing that handling of the uncertainty improves the precision of the a‐parameters. One case has a very small efficiency of .37 showing a high advantage for the uncertainty‐adjusted design: The third item in the three‐item block. This is a difficult item with a high discrimination ($a_{3}=2.5,b_{3}=2$). In that case of a steep item characteristic curve, relying on, possibly false, knowledge of the abilities makes the design bad if there is uncertainty (i.e. especially if m=6).

**TABLE 5:** Relative individual parameter c‐efficiency and D‐efficiency per item of D‐optimal design derived for known abilities versus an uncertainty‐adjusted D‐optimal design for $m\in \{6,30\},a_{op}=1$ and the item block examples in Section 4.2.

m	Optimality	Two‐item block		Three‐item block			Four‐item block			
		Item		Item			Item			
		1	2	1	2	3	1	2	3	4
6	a	.91	.91	.83	.88	.37	.61	1.06	1.06	.61
	b	1.16	1.16	1.22	1.07	.86	1.03	1.08	1.08	1.03
	D	.98	.98	1.04	1.03	.63	.83	1.07	1.07	.83
30	a	.95	.95	1.04	.91	.80	.80	1.03	1.03	.80
	b	1.06	1.06	1.06	1.02	1.01	1.03	1.03	1.03	1.03
	D	1.00	1.00	1.06	1.00	.92	.96	1.03	1.03	.95

In contrast to the a‐parameter, the b‐parameter precision is slightly worse in most cases when the uncertainty is handled. To explain this, we note that it is most informative for the difficulty parameter to observe examinees with ability around this difficulty level, whereas for a good estimation of the discrimination parameter, we need examinees with abilities more spread out. Therefore, the D‐optimal design for known ability has its drawback for the estimation of the discrimination parameter.

The different performance of the discrimination and the difficulty parameter in Table 5highlights an important consideration: the choice of the optimality criterion. The design is optimized for the D‐criterion, but we see that the difficulty parameter estimate is not improved. If the difficulty parameter is in focus of the calibration study, one needs to consider the parameter efficiency for this parameter and challenge if D‐optimality is the right choice (and alternatively utilize the c‐optimality criterion). However, we believe that the b‐parameter is usually not the only important quantity. For example, when making a decision about the use of items in the future, the test information is important which is the sum of item information (10). When we want to have an estimate of the item information, we need to estimate the discrimination parameter and the item characteristic curve $p_{i}(\theta )$. Optimizing the D‐criterion rather than just focusing on either a or b parameter also helps to estimate item information. An alternative criterion directly optimizing the integrated precision of the item characteristic curve is the so‐called I‐optimality (Bjermo et al., 2024). The I‐efficiencies can also be computed using the package optical.

### 5.5 Bias and MSE simulation results

The bias is shown in Table 6. We see in general that biases exist for all designs, but they are relatively small, within ±.2 for the discrimination and within ±.06 for the difficulty parameter. The values for the RD are in most cases a little closer to 0 compared with the two optimal designs.

**TABLE 6:** Bias for item parameters based on 1000 simulations for the uncertainty‐adjusted D‐optimal design (OD‐U), the D‐optimal design for known abilities (OD‐K), the RD for item block examples in Section 4.2, m=30 operational items, N=500 examinees.

Parameter	Design	Two‐item block		Three‐item block			Four‐item block			
		Item		Item			Item			
		1	2	1	2	3	1	2	3	4
a	OD‐U	−.10	−.09	−.03	−.19	−.15	−.05	−.02	−.03	−.08
	OD‐K	−.10	−.09	−.03	−.20	−.18	−.07	−.02	−.03	−.08
	RD	−.09	−.09	−.01	−.16	−.01	−.03	−.01	−.01	−.02
b	OD‐U	−.02	.02	−.06	.02	.04	−.04	−.01	.01	.05
	OD‐K	−.06	−.02	.02	.02	.05	−.04	−.01	.01	.06
	RD	−.02	.02	−.09	.01	.05	−.06	−.00	.02	.05

Table 7 shows the MSE. Note that the ratio of the item MSEs between designs is the empirical analogue to the item efficiency reported earlier. In general, we see similar tendencies in the ratio of these MSE values compared to the reported item parameter efficiencies in Tables 4 and 5. However, because the analytically based item efficiency corresponds to large sample sizes, the ratio for finite sample sizes might differ from the theoretical asymptotic efficiency.

**TABLE 7:** MSE for item parameters based on 1000 simulations for the uncertainty‐adjusted D‐optimal design (OD‐U), the D‐optimal design for known abilities (OD‐K), the RD for item block examples in Section 4.2, m=30 operational items, N=500 examinees.

Parameter	Design	Two‐item block		Three‐item block			Four‐item block			
		Item		Item			Item			
		1	2	1	2	3	1	2	3	4
a	OD‐U	.054	.052	.050	.096	.444	.094	.050	.054	.098
	OD‐K	.055	.054	.047	.103	.447	.119	.048	.053	.118
	RD	.073	.072	.063	.135	.776	.166	.061	.069	.146
b	OD‐U	.018	.018	.095	.016	.066	.066	.054	.056	.082
	OD‐K	.017	.018	.091	.015	.083	.073	.052	.055	.098
	RD	.026	.025	.208	.018	.260	.179	.055	.063	.144

## 6 DISCUSSION

Optimal calibration of pretest items generally assumes known examinee abilities. However, in a real testing situation these are unknown, and typically estimates based on an operational test would be used instead. We investigated two methods to deal with the fact that the abilities are estimated and incorporated the uncertainty of the estimates into the optimal item calibration methodology. One method approximates the ability posterior for every examinee with a normal distribution based on information from the operational items. The other method does not make this approximation. It is more robust to avoid the approximation. However, by making the approximation, we simplify the optimal design computation. Moreover, we have observed that the difference between the two methods was not that large. Therefore, the method of making the normal distribution approximation has an advantage.

An important difference between the two methods also has practical implications: If no distributional approximation is made, we first need to collect and analyse the results of the operational items for all examinees before we can calculate the optimal calibration design. This is possible if a separated set‐up is used with, e.g., some weeks between the operational test and the calibration test. In an integrated set‐up, however, this is hardly possible. When the distributional approximation is made and if we have reliable item parameters for the operational items available, the calculation of the optimal calibration design can be made before the integrated test.

We concluded that the values in the information matrix are almost the same with 100, 1000, or 10,000 posterior draws for the Monte Carlo approximation method. Sampling 100 instead of 10,000 posterior draws is about 100 times faster. Even though the posterior sampling can be done in a couple of seconds, calculating the optimal calibration design still needs to be done after analysing the results of the operational items, which constitutes the majority of the time it takes to calculate the design. We compared the information matrices for the whole ability span. In reality, we would need matrices for smaller intervals, which means that 100 posterior draws for the entire ability span would not be enough.

Because the calibration items are given at the end of the test when using the integrated test set‐up, the item parameter estimates can be affected by position effects (Leary & Dorans, 1985). A decline in examinee effort can result in a lower probability of a correct response of the items at the end compared to the items earlier in the test (Debeer et al., 2014; Nagy et al., 2019). It can result in an overestimation of the item's difficulty, which means that an item will seem harder than it is. The separated set‐up does not have the same problem. However, there is a general concern of conducting a separate calibration test if this is perceived as a low‐stake test by the examinees, e.g., by random responders (Van Laar & Braeken, 2022).

We have demonstrated that the optimal calibration design changes if it is acknowledged that abilities are estimates and not true values. The uncertainty‐adjusted optimal design is more robust around central ability intervals, but also gets less efficient when the uncertainty around the estimates becomes larger. However, the uncertainty needs to be handled in the optimal design approach; otherwise, the computed design would be incorrect. We have seen that naively using the method ignoring the uncertainty in abilities reduces the efficiency of the design when the uncertainty is large. With a larger size of the operational test, which is the basis for the ability estimates, the naive method has high efficiency.

In our investigations, we fixed the uncertainty in abilities by considering an operational test which has items with equidistant difficulties and all with the same discrimination. We do not claim that this is a realistic set‐up; in reality, the test would have items with some spread in discrimination and might have more easy items or more difficult items. However, our set‐up makes it possible to identify the important influence of increasing number of operational items without disturbance by differences in item parameters between different test sizes. Furthermore, we used sizes of operational tests down to six items. In reality, one would rarely have such small operational tests. However, realistic tests might exist having more items with lower discrimination, which yield the same uncertainty in abilities.

We are considering the uncertainty in ability estimates when deriving the optimal design, and the method for estimating the calibration item parameter is then open for choice. The OEM and MEM methods (Ban et al., 2001; Chen et al., 2012) consider the uncertainty during the estimation process. Another topic for future research would be to compare our design approach to estimating the calibration item parameters where uncertainty is considered in the estimation process, such as in the OEM and MEM methods. Or if a combination of the both would improve the result even further regarding item parameter recovery.

## AUTHOR CONTRIBUTIONS

**Jonas Bjermo:** investigation; writing – original draft; methodology; writing – review and editing; formal analysis. **Ellinor Fackle‐Fornius:** writing – original draft; validation; writing – review and editing. **Frank Miller:** investigation; funding acquisition; methodology; writing – review and editing; software; formal analysis; project administration; supervision.

## CONFLICT OF INTEREST STATEMENT

The authors declare no conflicts of interest.

## Data Availability

The code and data used for producing the results are available in the following GitHub repository: https://github.com/Bjermo/‐Optimizing‐calibration‐designs‐with‐uncertainty‐in‐abilities.
